# Key reaction components affect the kinetics and performance robustness of cell-free protein synthesis reactions

**DOI:** 10.1016/j.csbj.2021.12.013

**Published:** 2021-12-13

**Authors:** Alice M. Banks, Colette J. Whitfield, Steven R. Brown, David A. Fulton, Sarah A. Goodchild, Christopher Grant, John Love, Dennis W. Lendrem, Jonathan E. Fieldsend, Thomas P. Howard

**Affiliations:** a School of Natural and Environmental Sciences, Newcastle University, Newcastle upon Tyne NE1 7RU, United Kingdom; b Synthace Ltd., London W12 7FQ, United Kingdom; c Defence Science and Technology Laboratory, Porton Down, Salisbury SP4 0JQ, United Kingdom; d Biosciences, University of Exeter, Exeter EX4 4QD, United Kingdom; e Translational and Clinical Research Institute, Newcastle University, Newcastle upon Tyne NE1 7RU, United Kingdom; f Computer Science, University of Exeter, Exeter EX4 4QF, United Kingdom

**Keywords:** 3-PGA, 3-phosphoglyceric acid, ATP, adenosine triphosphate, cAMP, cyclic adenosine monophosphate, CFE, cell-free extract, CFPS, cell-free protein synthesis, CoA, coenzyme A, CTP, cytidine triphosphate, DoE, Design of Experiments, DSD, Definitive Screening Design, DTT, dithiothreitol, eGFP, enhanced green fluorescent protein, FEU, fluorescein equivalent units, G-6-P, glucose-6-phosphate, GTP, guanosine triphosphate, HEPES, 4-(2-hydroxyethyl)-1-piperazineethanesulfonic acid, K-glutamate, potassium glutamate, LB, lysogeny broth, Mg, magnesium glutamate, NAD, nicotinamide adenine dinucleotide, NTP, nucleoside triphosphate, OFAT, one-factor-at-a-time, PEG-8000, polyethylene glycol 8000, PEP, phosphoenolpyruvate, RFU, relative fluorescence units, RSM, Response Surface Model, tRNA, transfer ribonucleic acid, UTP, uridine triphosphate, X-gal, 5-bromo-4-chloro-3-indolyl-β-D-galactopyranoside, Automation, Cell-free protein synthesis (CFPS), Design of Experiments (DoE), Robustness, Statistical engineering

## Abstract

Cell-free protein synthesis (CFPS) reactions have grown in popularity with particular interest in applications such as gene construct prototyping, biosensor technologies and the production of proteins with novel chemistry. Work has frequently focussed on optimising CFPS protocols for improving protein yield, reducing cost, or developing streamlined production protocols. Here we describe a statistical Design of Experiments analysis of 20 components of a popular CFPS reaction buffer. We simultaneously identify factors and factor interactions that impact on protein yield, rate of reaction, lag time and reaction longevity. This systematic experimental approach enables the creation of a statistical model capturing multiple behaviours of CFPS reactions in response to components and their interactions. We show that a novel reaction buffer outperforms the reference reaction by 400% and importantly reduces failures in CFPS across batches of cell lysates, strains of *E. coli*, and in the synthesis of different proteins. Detailed and quantitative understanding of how reaction components affect kinetic responses and robustness is imperative for future deployment of cell-free technologies.

## Highlights

• Novel cell-free protein synthesis reaction buffer improves performance by 400%.
• Enhanced performance is maintained across the synthesis of different proteins.
• Protein synthesis performance is robust across different cell lysate batches and *E. coli* strains.
• Buffer components affect aspects of reaction kinetics in differing ways.

## Graphical abstract

**Figure:**
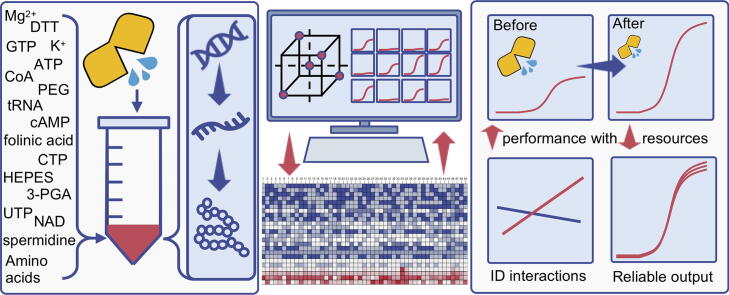

## 1 Introduction

Cell-free systems have established themselves as key platforms for the delivery of synthetic biology projects. Their applications include the production of therapeutics [1], pharmaceuticals [2] and viral particles for vaccine development [3]; characterisation and prototyping platforms for genetic diseases [4]; and as biosensors for medical diagnostics (e.g. the detection of Zika and Ebola viruses [5]) or environmental monitoring (e.g. the detection of metals, antibiotics and aromatic compounds [6]). A key appeal of cell-free systems is the open nature of the reactions which allows a high degree of control over reaction conditions and permits manipulations that would not be viable in living cells. Additionally, cell-free synthetic biology possesses the distinct advantage of not needing to trade off the demands of a living cell against user-defined goals. This can allow greater conversion efficiencies of substrate to product (e.g. [7]), and provide a route to the synthesis of industrially relevant compounds such as styrene and 2, 3-butanediol, which are toxic to cell growth [8], [9]. In addition, cell-free systems allow for the inclusion of non-canonical amino acids to engineer diverse protein chemistries [10].

One of the most popular cell-free methods is the use of either cell lysates or purified proteins to conduct transcription and translation reactions of designed genetic constructs outside of a living cell, also known as cell-free protein synthesis (CFPS). These reactions are of particular interest for applications such as gene construct prototyping, biosensor deployment beyond the laboratory and the production of proteins with novel chemistry. Protocols for the preparation of cell lysates (or cell-free extracts, CFEs) for these reactions can be complex, prescriptive and time consuming, while reaction buffers comprise many components, several of which can be expensive. As such, there has been significant effort to optimise these protocols. Optimisation goals include increasing protein yield [11], [12], improving reaction reproducibility [13], [14], decreasing CFE preparation time [15], [16], [17], [18] and reducing protocol costs [19], [20], with efforts typically focussing on a single stage of the protocol, such as cell growth, cell lysis [17], [21], the inclusion of a run-off reaction [22] or the composition of the reaction mixture itself. To quantify the performance of CFPS reactions the signal from a reporter protein or the yield of a protein is typically measured, though often as a single endpoint measurement [23]. This approach can provide valuable information relevant to protein yield; but is of less value for probing the kinetic responses of the system. Moreover, the behaviours of multiple kinetic responses (e.g., rate of reaction or reaction lag time) are not commonly investigated but are highly relevant to the development of diagnostic devices.

A further challenge to the study of CFPS is the extent of the design space. With numerous factors impacting on the kinetics of the reactions, this is difficult to explore using one-factor-at-a-time (OFAT) experimental approaches. Design of Experiments (DoE), by contrast, provides the means to efficiently explore complex design spaces in a meaningful way. By sampling a subset of experimental settings from within a multidimensional design space, and performing iterative experiments guided by initial learning, it is possible to understand the impact of key components as well as factors which interact to impact on performance. Additionally, by considering multiple responses relating to performance kinetics, for example yield, rate and longevity, it is possible to study multiple responses simultaneously and therefore to identify response trade-offs and system limitations. This systematic approach has previously been applied to optimise and understand various bioprocesses ranging from the optimisation of recombinant antibody production [24] and improving metabolic pathway efficiency [25], to modelling the ethanol biosynthetic pathway in yeast [26] and developing high-performance whole cell biosensors [27]. Combining laboratory automation with statistically-structured experimental design serves as an efficient means to obtain highly informative datasets that can be used to model complex systems. Such approaches have been used to build predictive models to maximise protein production in cell-free systems [23], [28] but in each case the optimisation was not focussed on reaction kinetics directly. Moreover, where different classes of reaction kinetics were observed [28], this could primarily be attributed to changes in gene constructs rather than buffer composition.

Here, we use systematic experimentation to examine how the stages of CFE preparation and different reaction components impact on key aspects of the kinetics of CFPS reactions. We investigated three stages of CFE preparation: (i) cell growth, (ii) cell lysis and (iii) extract clarification as well as (iv) examining the chemical composition of the CFPS reaction buffer. We quantified the impact of these factors by characterising CFPS reactions in terms of the peak response, the maximum reaction rate, the time to reach the maximum rate (lag time), and the longevity of the reaction. We employed DoE to examine the impact of multiple factors simultaneously and in an efficient manner. Furthermore, this approach allowed us to identify interacting factors having an impact on performance which would not be possible using traditional sequential experimentation. This methodical approach has led to the development of a novel CFPS reaction buffer, outperforming the reference reaction by 400%, while maintaining a robust performance with different batches of CFE, in the synthesis of different proteins, and using CFE derived from an alternative strain of *E. coli*.

## 2 Materials and methods

### 2.1 Fluorescein standards

Each experimental 384-well microplate included a set of 12 fluorescein standards at 100 µL. The Fluorescein NIST-Traceable Standard (F36915) was used at a concentration range from 0 to 400 nM at two-fold dilutions using 50 mM glycine-NaOH (pH 9.5) as a diluent.

### 2.2 Fluorescence data collection

Reactions containing a DNA template coding for a fluorescent reporter were performed in black 384-well flat-bottomed microplates. CFPS reactions and fluorescein standards were incubated using a Varioskan LUX Multimode Microplate Reader (Thermo Scientific). A kinetic loop was used to record top-down fluorescence readings at 5 min intervals for a total of 12 h with continuous shaking at 60 rpm and incubation at 37 °C. Fluorescein and eGFP were monitored using an excitation of 488 nm and emission of 512 nm and mCherry with an excitation of 587 nm and emission of 610 nm. An excitation bandwidth of 5 nm and measurement time of 100 ms was used for all readings. Data collection for a subset of experiments (DSD1 and DSD2) was performed using a CLARIOstar (BMG LABTECH). Fluorescence readings were recorded at 5 min intervals for a total of 12 h with continuous shaking at 100 rpm and incubation at 37 °C. Fluorescein and eGFP were monitored using an excitation of 488 nm and emission of 521 nm with an excitation bandwidth of 8 nm and measurement time of 100 ms. A Breathe-Easy® sealing membrane (Sigma-Aldrich) was used to prevent evaporation in the CLARIOstar and bottom-up readings were taken to avoid interference from condensation.

### 2.3 Experimental design and statistical analysis

JMP Pro 13 (SAS) was used to generate statistically structured arrays of experimental runs, using the Design of Experiments (DoE) function, and to perform statistical analyses. Linear regressions were performed using Prism8 (GraphPad).

### 2.4 Cell-free extract preparation

Batches of CFE were prepared based on the protocol described by Sun et al. [29] with some modifications. Briefly, a 1 L culture of *E. coli* was grown to a high cell density. Cells were harvested and washed in S30A buffer, and the pellets flash frozen in liquid nitrogen and stored at −80 °C. Cell pellets were resuspended in S30A buffer at a 1:1.2 g:mL ratio and sonicated for a total of 5 min at 120 W, 20 kHz, 30% amplitude at 20 s/40 s on/off cycles. Cell debris was removed by centrifugation and the supernatant incubated at 37 °C for 1 h at 200 rpm. A centrifugation step removed residual cell debris and the supernatant was dialysed in S30B buffer for 2 h at 4 °C. A final centrifugation step was used to clarify the CFE before aliquoting for storage at −80 °C. Protein concentration was determined by NanoDrop™. Cell lysates for use in Scoping trials were prepared as described in Appendix Method A1 and Appendix Table A1.

### 2.5 Plasmid assembly and preparation

The plasmids pTU1-A_J23100_pET-RBS_eGFP_BBa_B0015 and pTU1-A_J23100_pET-RBS_mCherry_BBa_B0015 were assembled using parts available in the EcoFlex MoClo Kit [30]. Plasmids housing the required bioparts were extracted using a QIAGEN Plasmid Mini Kit. Reactions included 20 U BsaI-HF (New England Biolabs), 1–3 U T4 DNA Ligase (Promega), 10× ligase buffer (Promega), 1 mg/mL BSA, 50 ng plasmid backbone, 100 ng each insert, ddH_2_O to 15 µL total volume. Reactions were incubated at 37 °C for 5 min and 16 °C for 10 min for 15 cycles, followed by 50 °C for 5 min and 80 °C for 5 min. 5 µL reaction mix was used to transform 25 µL One Shot TOP10 Chemically Competent *E. coli* (ThermoFisher Scientific) and cells were spread onto selective LB agar containing 20 mg/mL X-gal for blue/white screening. Plasmids were sequence verified by Eurofins Genomics using the primers pTU1-A-seq_Fwd (5′-GGAATTCGCGGCCGCTTCTAGAA-3′) and pTU1-A-seq_Rvs (5′-AGCGAGTCAGTGAGCGAGGAAG-3′). The pTU1-A-lacZ plasmid was used directly from the EcoFlex MoClo Kit without modification. Large-scale plasmid extractions for use in CFPS reactions were performed using the QIAGEN Plasmid Maxi Kit.

### 2.6 Cell-free protein synthesis reactions

All reactions were performed at 100 µL volumes in black 384-well flat-bottomed microplates unless otherwise stated. Reactions were incubated at 37 °C for 12 h with gentle shaking. Reference reaction conditions were: 8.9 mg/mL CFE, 5 mM Mg-glutamate, 120 mM K-glutamate, 1.5 mM each amino acid except leucine, 1.25 mM leucine, 50 mM HEPES, 1.5 mM ATP, 1.5 mM GTP, 0.9 mM CTP, 0.9 mM UTP, 0.2 mg/mL tRNA, 0.26 mM CoA, 0.33 mM NAD, 0.75 mM cAMP, 0.068 mM folinic acid, 1 mM spermidine, 30 mM 3-PGA, 2% PEG-8000, 20 mg/mL pTU1-A_J23100_pET-RBS_eGFP_BBa_B0015. Experimental reaction compositions are described in Source Data files. Additional plasmids used in experiments to test buffer robustness between DNA templates were pTU1-A_J23100_pET-RBS_mCherry_BBa_B0015 and pTU1-A-lacZ. Reactions containing pTU1-A-lacZ were performed in clear 384-well flat-bottomed microplates and also included X-gal at 2 mM, absorbance at 650 nm was recorded at the end-point of the reaction.

### 2.7 Automation

The set-up of combinatorial experimental arrays was facilitated by automated liquid handling. The epMotion® 5073 m (Eppendorf) was used for all experiments with the exception of DSD1 and DSD2 where the dragonfly discovery (TTP Labtech) was used to reduce set-up time. The DSDs were designed using JMP Pro 13 (SAS) and the designs translated to liquid handling instructions for the dragonfly discovery using the Antha platform (Synthace Ltd.). See Appendix Method A2 for detailed methodology and Source Data files for experimental design and execution files containing parameter set points.

## 3 Results

### 3.1 Concentrations of Mg-glutamate and K-glutamate, rather than lysate preparation method, have the greatest impact on the reaction kinetics of cell-free eGFP synthesis

**Fig. 1:**
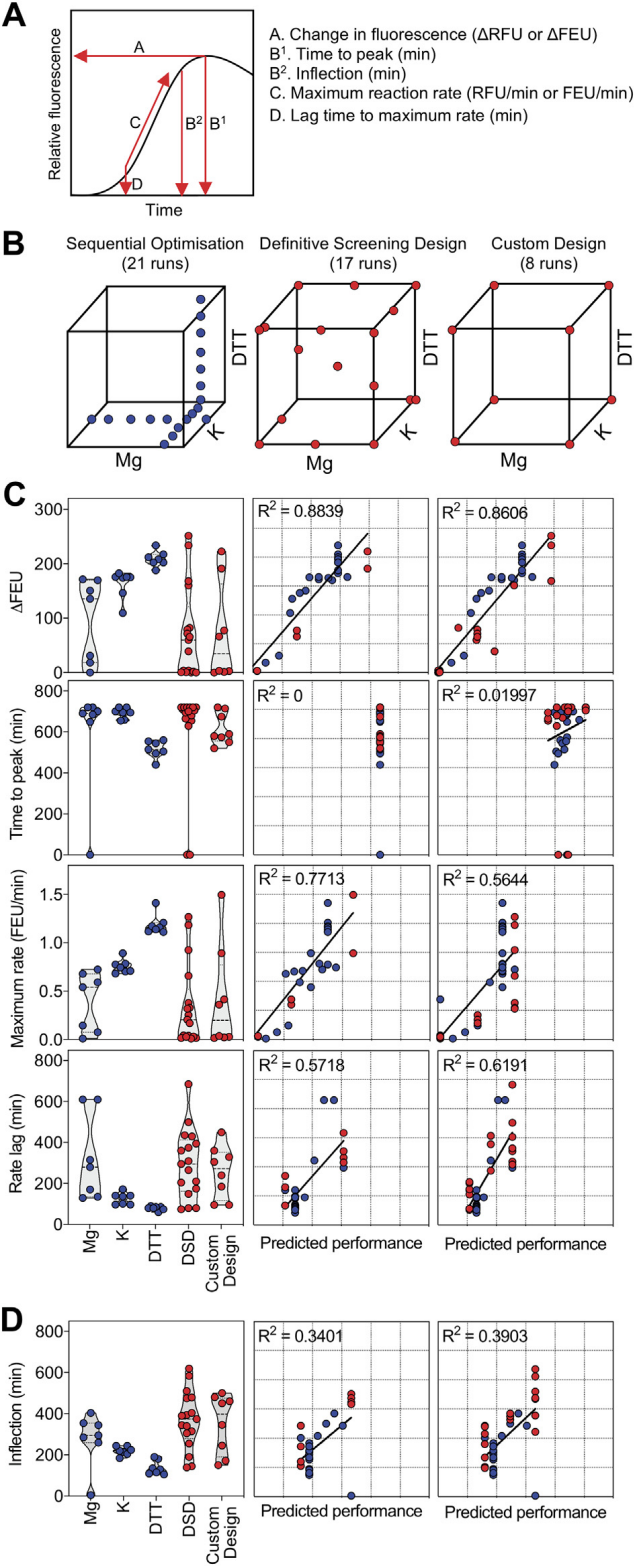
Impact of Mg-glutamate, K-glutamate and DTT on CFPS reaction kinetics. A) Measurements extracted to characterise CFPS performance: A. Change in fluorescence (ΔFEU) B^1^. Time to peak (min) B^2^. Inflection (min) C. Maximum reaction rate (FEU/min) D. Lag time to maximum rate (min). B) Experimental design spaces covered by the recommended 21-run sequential optimisation vs. 17-run Definitive Screening Design and 8-run DoE Custom Design. C) Extracted characterisation data indicating ΔFEU, time to peak, maximum rate and rate lag from comparative designs (left). Predictive capability of Least Squares models constructed using DSD data and validated using unseen data from Sequential Optimisation and Custom Design (centre). Predictive capability of Least Squares models constructed using Custom Design data and validated using unseen data from Sequential Optimisation and DSD (right). D) Extracted inflection data from comparative designs (left). Predictive capability of Least Squares model constructed using DSD data and validated using unseen data from Sequential Optimisation and Custom Design (centre). Predictive capability of Least Squares model constructed using Custom Design data and validated using unseen data from Sequential Optimisation and DSD (right). See Fig. 1 Source Data.xlsx for reaction compositions and responses.

We wished to determine how cell lysate (cell-free extract, CFE) preparation and the concentration of components in cell-free protein synthesis reactions impact on the kinetics of cell-free protein synthesis (CFPS). We used a constitutive eGFP expression cassette that allowed the collection of data continuously and from many experiments simultaneously. Cognisant that it was important to be able to compare values across different experimental blocks, a dilution series of the Fluorescein NIST-Traceable Standard was included alongside each experiment (Appendix Fig. A1). Kinetic responses were therefore converted to relevant kinetic parameters before undergoing further statistical analysis. Reactions were performed for 12 h at 37 °C and time course data were smoothed using a weighted sliding window of size five, to extract all of the relevant kinetic parameters. Specifically, the smoothed datum at time point *t* is the average of a weighted combination of the raw datum at *t* (weight 2) plus the raw observations at *t + 1* and *t* − *1* (weighted at 1 each) and those at *t + 2* and *t* − *2* (weighted at 0.5 each). These parameters were: A. the change in fluorescence (ΔFEU), B. the maximum rate at which the fluorescence signal increased, C. the time taken to reach the maximum rate (or lag time) and D. the time taken for the reaction to peak (time to peak) (Fig. 1A). We divided our protocol for lysate based CFPS into four phases (Fig. S1, Appendix Method A1, Appendix Table A1). The first phase covers the growth and harvest of *E. coli* cells; the second covers cell lysis; the third stage covers CFE clarification; the fourth and final component of the protocol represents the composition of the reaction buffer. This comprises amino acids, NTPs, cofactors, energy regeneration components, molecular crowding agents, buffering agents and the supplied DNA template [29]. In agreement with work from Failmezger et al. [31], our data indicated that while it is important to grow cells to a moderate to high cell density, leaving cells in stationary phase was not detrimental to CFPS reactions (Fig. S1). These trials also confirmed previous analysis that sonication is a more effective method for generating functional CFEs than bead beating [32]. Both moderate and high sonication conditions resulted in a strong CFPS performance, though extracts produced in moderate conditions were slower to reach maximum protein synthesis rates. Regarding the third phase, extract clarification, we observed that the most extreme settings resulted in poor extract performance, consistent with previous studies that argue for this extract clarification stage [33], [34]. However, removing this step from the protocol, while possible, comes with a clear impact on performance. Finally, data from trials in which the reaction composition itself was altered demonstrated that the four response criteria were affected by these changes and that changes in composition did not impact equally on each response. For example, high concentrations of reaction components resulted in faster production of a fluorescence signal but not a higher overall fluorescence signal. We therefore focussed on exploring how reaction components affect reaction kinetics whilst maintaining cell growth, disruption and clarification methods at settings shown not to impair CFPS reaction performance.

Many protocols for CFPS recommend optimisation of Mg-glutamate for every unique CFE preparation [35], [36], [37]. Other CFPS protocols also include K-glutamate and DTT in the list of components that should be calibrated for a given CFE batch [29]. We compared the efficiency of optimising *via* a commonly employed sequential, one-factor-at-a-time (OFAT) approach against two statistically designed experimental arrays, a Definitive Screening Design (DSD) and a Custom Design (Fig. 1B). For the sequential approach, experiments were first conducted at seven different Mg-glutamate concentrations. The results demonstrated that concentrations >4 mM Mg-glutamate achieved the greatest ΔFEU, and that the highest Mg-glutamate concentrations (5 mM and 6 mM) achieved the fastest rates. Next, experiments were performed using seven different K-glutamate concentrations with the Mg-glutamate concentration fixed at 5 mM. The results indicated that a minimum of 60 mM K-glutamate is required for a strong performance, with 120 mM giving the best response. In the final phase (5 mM Mg-glutamate, 120 mM K-glutamate) it was observed that a DTT concentration between 1 and 2 mM resulted in high ΔFEU and the fastest rates. We compared these conclusions to data generated using two separate multifactorial experiments. Without optimisation, experimental reaction compositions included in the eight-run Custom Design and the 17-run DSD reaction mixtures sustained equivalent or higher ΔFEU and maximum rates than were observed in the 21-run sequential optimisation (Fig. 1C, left). For the statistical analysis, data for all four responses (Fig. 1A) from the 17-run DSD were analysed using a Fit Definitive Screening and the eight-run Custom Design using a Fit Two Level Screening method. The results highlighted Mg-glutamate and K-glutamate were significant factors with respect to the ΔFEU and both analyses indicated a potential interaction between Mg-glutamate and K-glutamate; specifically, at high concentrations of Mg-glutamate the effect of K-glutamate on ΔFEU is positive, while at low concentrations of Mg-glutamate, K-glutamate may have a neutral or negative effect. The same main effects and interaction were observed with respect to the reaction rate when analysing the DSD, however, the analysis of the smaller Custom Design only identified Mg-glutamate, and not K-glutamate, as having a positive impact on reaction rate. Analysis of the DSD did not identify any factors significantly impacting on the time taken to reach peak fluorescence. Conversely, analysis of the Custom Design suggested a potential antagonistic interaction between K-glutamate and DTT, whereby increased DTT results in a longer time to peak, but only at elevated levels of K-glutamate. Finally, the results from both the DSD and Custom Design indicate that increasing Mg-glutamate concentration may reduce the time taken to reach the maximum reaction rate. DTT was not found to be a significant factor for any response, though it may interact with other components of the reaction mixture.

Using terms identified as important for model projection from the DSD, we built Least Squares models for each response. We used the 29 measured values for the sequential OFAT and Custom Design experiments (i.e., unseen values not used in model construction) to validate these models. The Least Squares models using only Mg-glutamate and K-glutamate as factors explain 88.4% of the variation in ΔFEU, 77.1% of the variation in rate and 56.4% of the variation in rate lag (Fig. 1C, centre). Least Squares models were also created using data collected from the eight-run Custom Design and validated by plotting model predictions against the 38 observed responses from the OFAT and DSD. These models – with only Mg-glutamate and K-glutamate as factors – were able to explain 86.1%, 61.9% and 56.4% of the variation for ΔFEU, rate and lag respectively (Fig. 1C, right). The Least Squares models based on time to peak measurements were unable to predict the performance of unseen experimental combinations (Fig. 1C, second row). The measurement extraction was therefore revised to indicate the inflection point at which the rate of reaction slowed, as opposed to the point at which the maximum response was reached. The inflection point was calculated by first taking the first difference of the moving-window smoothed data series, and then marking the timepoint where the mean of the cumulative of this time series is maximised. This can be used as a metric to indicate the longevity of a reaction. Using the inflection point as an indicator of longevity, Least Squares models constructed using data from the DSD and Custom Design respectively were able to explain 34% and 39% of the observed variation in inflection point (Fig. 1D). All experiments from this point onwards used the inflection point to analyse longevity rather than the time to peak. Importantly, the outcome of the 17-run DSD and the eight-run Custom Design indicated the best conditions are to maintain high Mg-glutamate and high K- concentrations. This agrees with the 21-run, sequential OFAT experimental protocol while generating additional information on potentially interactions and requiring less time and fewer resources.

### 3.2 Nine components within the reaction composition have significant impact on CFPS kinetic parameters

**Fig. 2:**
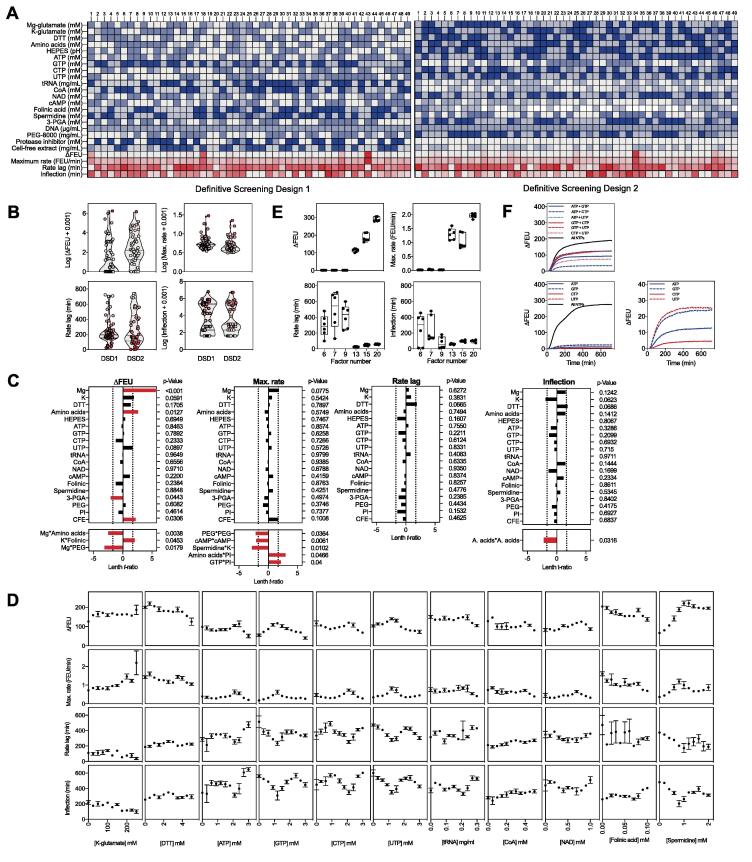
Screening the effects CFPS reaction components on reaction kinetics. A) Two 49-run DSDs to investigate 20 factors in CFPS reactions. Colour intensity is indicative of increased concentration (blue) or preferred response (red). B) Extracted, transformed measurements for DSD1 and DSD2. See Fig. 2A and B Source Data.xlsx for reaction compositions and responses. C) Fit Two Level Screening analysis to identify main factor effects impacting on responses. D) Individual drop-out tests to screen for essentiality and dose-dependent effects in factors showing a non-significant effect on any response. n = 3; error bars indicate standard error (s.e.m). See Fig. 2D Source Data.xlsx for reaction compositions and responses. E) CFPS responses in minimised reaction buffers by removing non-essential components. n = 6; error box indicates 25th–75th percentiles, whiskers indicate minimum and maximum responses. See Fig. 2E Source Data.xlsx for reaction compositions and responses. F) CFPS performance with reaction buffers containing one or two NTPs only, response presented represents mean response of six replicates. See Fig. 2F Source Data.xlsx for reaction compositions and responses. (For interpretation of the references to colour in this figure legend, the reader is referred to the web version of this article.)

Our initial experiments looked broadly at each of the three lysate preparation steps and at only three components of the cell-free reaction mixture. However, the CFPS reaction composition used here, based on the highly cited and widely used protocol published by Sun *et al* [29], consists of 38 components: Mg-glutamate, K-glutamate, DTT, 20 different amino acids, HEPES, ATP, CTP, GTP, UTP, tRNA, CoA, NAD, cAMP, folinic acid, spermidine, 3-PGA, PEG-8000, DNA template and CFE. Moreover, we included a protease inhibitor in the reaction mix to test whether an observed decrease in fluorescence observed during the later stages of some CFPS reactions was the result of protein turnover. Our first goal was to experimentally reduce the number of factors under investigation. This may be achieved by either setting a concentration at which they may be held constant while key factors are investigated or preferably by removing reaction components completely. We grouped each of the 20 amino acids as a single factor, reasoning that the requirement for amino acids would vary depending on the protein being synthesised, and therefore optimising amino acid ratios for one protein would unlikely be optimal for another. The DNA concentration was fixed at 60 mg/mL in all experiments unless stated otherwise. A review of CFPS protocols identified differences in the concentrations of each component used in reaction compositions (Table S1) and these outer limits guided the range of concentrations used in a second, larger DSD than used previously (DSD1). The centre point concentrations were maintained at the levels described by Sun et al. [29], using concentrations of Mg-glutamate, K-glutamate and DTT determined by the sequential optimisation. DSD1, comprising 49 runs, was performed as previously described and the ΔFEU, maximum rate, rate lag and inflection values were extracted for each reaction. The data for ΔFEU, maximum rate and inflection were right-skewed and were therefore log transformed prior to analysis. While some of the CFPS experimental compositions from DSD1 were successful, most of the reactions performed poorly indicating our factor ranges were set too broadly (Fig. 2A). To address this, the factor ranges were refined based on an analysis of compositions achieving both a high ΔFEU and maximum rate. The refined factor ranges used the best performing reaction settings from DSD1 as the new centre point settings. The protease inhibitor was removed from future reactions as its addition had a consistent negative impact on CFPS performance (Fig. S2A). A further 49-run DSD was performed (DSD2) with a greater proportion of successful reactions compared to DSD1 (Fig. 2B). The results of both 49-run DSDs were analysed together using a two-level screening model (Fig. 2C). This analysis identified reaction components and significant interactions between components that had positive and negative impacts on each response. Concentrations of Mg-glutamate, amino acids and CFE were found to have a significantly positive effect on ΔFEU. Furthermore, the effect of Mg-glutamate on ΔFEU was found to be affected by interactions with amino acids and PEG-8000. 3-PGA was identified as having a negative impact on ΔFEU and the maximum rate. Regarding maximum rate, both PEG-8000 and cAMP significantly affected this response, but the analysis detected curvature in the response suggesting a moderate setting may be optimal. Curvature was also detected regarding the effect of amino acids on the longevity of reactions, again suggesting a moderate setting may be favourable. HEPES was not found to have a significant effect on CFPS performance and reactions worked well across the range from pH 7.5–9 (Fig. S2A). The pH was therefore fixed at pH 8.0 for all subsequent experiments. We next tested whether components that were not statistically significant, or had negative effects, could be removed from the reaction mixture. These were K-glutamate, DTT, ATP, GTP, CTP, UTP, tRNA, CoA, NAD, folinic acid and spermidine. Reaction composition and concentrations were fixed at the reference settings [29] and each of the components in turn were removed from the reaction completely, or included at a range of concentrations that increased beyond the original DSD concentrations. Surprisingly, the results indicated that each of these eleven components could be removed completely from the reaction composition without affecting the CFPS of eGFP (Fig. 2D). In only one instance was there an impact of reducing concentration; spermidine concentrations below 1 mM resulted in 3-fold decrease in eGFP signal, though even in the absence of spermidine, eGFP fluorescence was observed. These results indicate that for these reaction components there is either a sufficient residual concentration in the lysate for the reactions to occur, or their function is provided by another residual component of the lysate.

With this in mind, we tested if minimal or reduced buffer compositions remained viable for protein synthesis. We tested a minimal CFPS buffer, containing only those four components that were deemed to have a significant positive impact on performance (Mg-glutamate, amino acids, PEG and CFE) along with a HEPES buffer and DNA. This minimal buffer was tested with and without spermidine. A nine-factor buffer was tested which contained each of these components, plus K-glutamate and DTT, as well as two further buffers which included all four NTPs (13-factor) and NAD and cAMP (15-factor). Finally, the full 20-factor reaction buffer was used as a control. Concentrations followed those described previously [29]. Our data indicated that CFPS reactions were not viable when all four NTPs were removed simultaneously despite it being possible to remove individual NTPs without reducing CFPS performance (Fig. 2E). We carried out supplementary tests to investigate the effect of including paired or individual NTPs only (Fig. 2F). We observed that CFPS was possible when a single NTP was included, however ΔFEU was reduced to <10% reference levels (mean ΔFEU = 17.14). CFPS reactions improved with two NTPs (mean ΔFEU = 98.06), but the performance was strongest when all four were included (mean ΔFEU = 277.23). This suggests that either some carry over of NTPs occurs within the lysate, or there is some capacity for interconversion of NTPs within the cell lysate [38]. Regardless, as NTP requirement will vary depending on the template DNA, the decision was made to include all four NTPs at a fixed setting in subsequent experiments. With regards to the other reaction components, CFPS is possible with only 13 components, but performance is reduced. When NAD and cAMP are included (15 components) the ΔFEU is two-fold greater, but it is noticeable that the maximum rate is not significantly different between the 13- and 15-component mixtures. Finally, when tRNA, CoA, folinic acid and 3-PGA are included both the ΔFEU and the rate improve despite the initial screening indicating that tRNA and CoA had no significant impact, while folinic acid and 3-PGA were found to have negative effects (with folinic acid interacting with both K-glutamate (significantly) and 3-PGA (weakly)). It is possible that the negative impacts of folinic acid and 3-PGA alone are counteracted by the positive interactions that folinic acid has with 3-PGA and K-glutamate. The data therefore suggest that these components should be included in the reaction buffer and require further investigation. Nine factors were therefore selected: Mg-glutamate, amino acids, NAD, cAMP, folinic acid, spermidine, 3-PGA, PEG-8000 and CFE. The remaining factors were included but at fixed concentrations except for the protease inhibitor which was removed entirely.

### 3.3 Complex interactions between reaction components necessitate fine tuning of concentrations to meet multiple response objectives

**Fig. 3:**
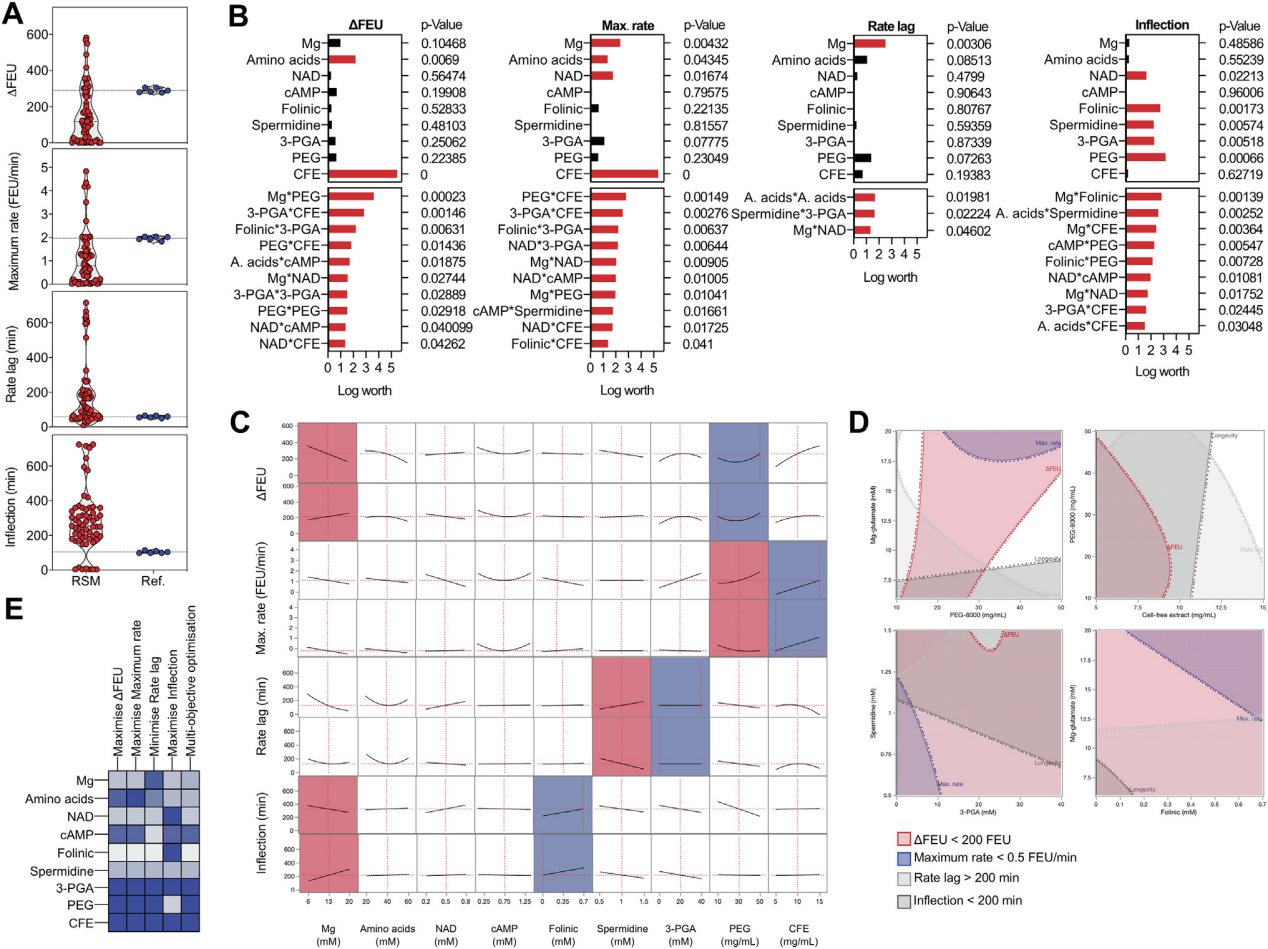
9-factor Response Surface Model indicating main factors and interactions affecting CFPS reaction performance. A) Violin plots showing the spread of responses obtained in a 9-factor RSM compared to the reference reaction. The dotted line indicates mean response of the reference reaction replicates. See Fig. 3 Source Data.xlsx for reaction compositions and responses. B) Single factor significance and significant two-way interactions impacting CFPS reaction kinetics as determined in a Least Squares model. All interactions deemed important in the model are presented in Fig. S3. C) Prediction profiler showing the key two-way interaction influencing each response. Vertical red dashed lines indicate the set-point for each factor. Factors shaded in blue are varied and factors shaded in red are influenced by altering the first variable. All other factors remain constant. D) Contour plots to illustrate key two-way interactions described in the Least Squares model. Factors are set at the reference reaction settings. Dots sit in the direction indicating an increased response. Shaded zones represent a less favourable response. White regions represent the design space remaining where factor settings must fall to balance the interaction and achieve a desirable response. E) Predicted best settings for single and multi-objective optimisation. Dark blue is indicative of an increased concentration. Concentrations are available in Table S1. (For interpretation of the references to colour in this figure legend, the reader is referred to the web version of this article.)

To explore how concentrations of nine components of the CFPS reaction affect four kinetic responses, we adopted a 61-run experimental array permitting a nine-dimensional Response Surface Model (RSM). Concentrations of the components were based on the data from the screening phase rather than the reference composition. The goal of this exercise was to build a statistical model that reflects the impact of the main factors on reaction kinetics as well as any higher order interactions. In addition, the RSM can be used to examine which concentrations represent a good trade-off between the different objectives. Of the 61 experimental compositions, 20% of the reactions (12/61 runs) gave performances that exceeded that of the reference composition (Fig. 3A). These reactions achieved a higher ΔFEU, a high reaction rate, greater longevity and a short lag time to reach maximum rate. Least Squares analysis was used to identify the main factors and two-way interactions impacting on CFPS performance (Fig. 3B, Fig. S3). CFE, 3-PGA, PEG-8000 and Mg-glutamate were identified as the factors having the greatest positive influence on performance (i.e., ΔFEU, maximum rate, lag time, longevity). Key two-way interactions that were identified in the RSM include Mg-glutamate*PEG-8000, 3-PGA*CFE, folinic acid*3-PGA and PEG-8000*CFE. The interaction between PEG and Mg-glutamate is identified in both the original DSDs and RSM analysis as one of the main interactions impacting on ΔFEU. Here, the RSM provides greater clarity about the nature of this interaction: if the concentration of PEG-8000 is high, increasing Mg-glutamate concentrations have a negative effect on ΔFEU; by contrast, if the PEG-8000 concentration is low, increasing Mg-glutamate concentrations have a positive impact on ΔFEU (Fig. 3C, top two rows). Regarding the rate of reaction, the model indicates that high concentrations of PEG-8000 may only be beneficial when the amount of CFE included is also high (Fig. 3C, second two rows). By contrast, if the amount of CFE included in the reaction is low, then increasing concentrations of PEG-8000 will have minimal impact or may even reduce the rate of reaction. Regarding the reaction lag time, the RSM model also identifies an interaction between 3-PGA and spermidine (Fig. 3C, penultimate two rows): here if the concentration of 3-PGA is high, increasing concentrations of spermidine increase the lag time, but when 3-PGA concentrations are low, increasing spermidine concentrations decrease the lag time. In terms of longevity, the model indicates that when folinic acid is included at a high concentration, increasing Mg-glutamate will have a negative impact, but when folinic acid concentrations are low, increasing Mg-glutamate concentration will increase the reaction longevity (Fig. 3C, bottom two rows). Finally, both folinic acid and 3-PGA have previously been identified as being important components for CFPS reactions (Fig. 2E) but there is also evidence of neutral or even negative impacts on CFPS performance (Fig. 2C, D, Fig. S2B). The RSM allows us to understand the interactions with greater clarity. In this instance CFPS reactions require either folinic acid concentrations to be low, when 3-PGA concentrations are high or folinic acid concentrations must be high, if 3-PGA concentrations are low. In scenarios where both are high, or both are low, the outcome is a reduced FEU.

To visualise how these interactions affect each of the response variables at the same time, four contour plots were constructed in which all reaction concentrations are set to the reference composition (Fig. 3D). In each case, the threshold contour for a desirable CFPS response was set at a minimum ΔFEU of 200, a minimum rate of 0.5 FEU/min, a maximum lag time of 200 min, and a minimum longevity of 100 min. The operating region where combinations meet all four of these criteria are shown as a white zone. At the reference reaction composition, the interaction between Mg-glutamate and PEG-8000 provides a small range of concentrations at which all three objectives are satisfied (i.e., where the PEG-8000 concentration is between 30 and 50 mg/mL and Mg-glutamate concentration is between 8 and 16 mM (the white zone)). Moreover, while there is a degree of flexibility awarded by the relationship between the amount of CFE and PEG-8000 (provided the amount of PEG-8000 is kept above 20 mg/mL), with this reaction composition it is not possible to achieve a balance between 3-PGA and spermidine or folinic acid and Mg-glutamate concentrations that meets all three criteria. As a result, reactions were proposed that either optimised single or multiple objectives (Fig. 3E). The predicted reaction compositions to maximise ΔFEU and reaction rate were similar to each other, but the reaction proposed to minimise lag time required a higher Mg-glutamate concentration, and lower amino acids and cAMP, and the reaction proposed to maximise longevity required higher concentrations of NAD and folinic acid and a lower concentration of PEG-8000. A composition predicted to balance the four objectives should contain Mg-glutamate concentrations below 12 mM, PEG-8000 above 40 mg/mL and CFE above 12 mg/mL. This reaction composition is similar to reaction 33 from the 61-run RSM array.

### 3.4 Reaction mixtures that minimise the amount of cell lysate create more robust environments for different preparations of cell extract

**Fig. 4:**
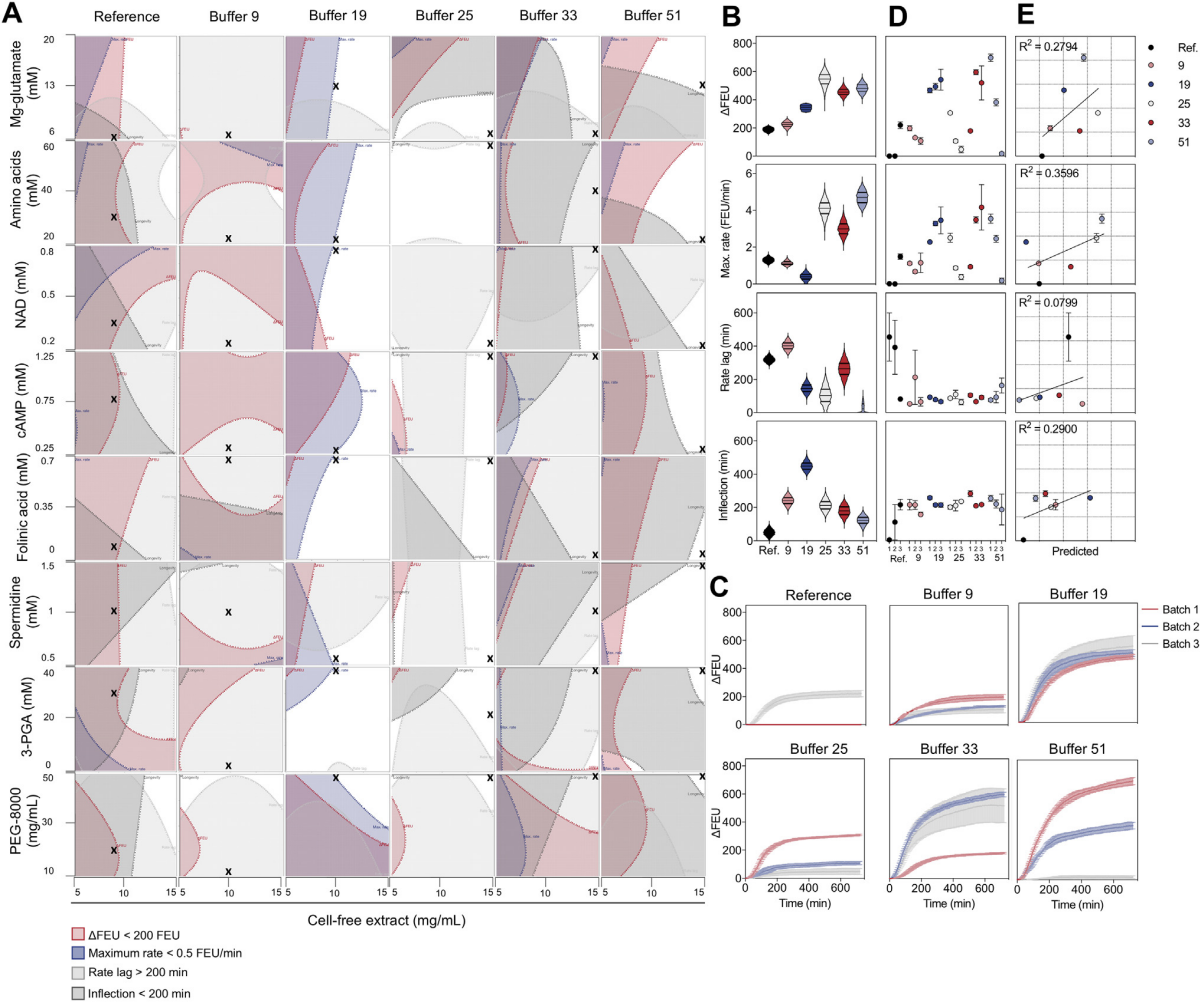
Reaction buffer robustness between independently prepared CFE batches. A) Contour plots to visualise key interactions between CFE and other reaction components in different reaction buffers, as described in the Least Squares model. Dots sit in the direction indicating an increased response. Shaded zones represent a less favourable response. White regions represent the operating space remaining where factor settings must fall to balance the interaction and achieve a desirable response. Black crosses indicate settings for the relevant buffer composition. B) Simulated responses of 5000 replicates allowing up to ±10% accuracy in component addition. C) Time-course data of CFPS reactions expressing eGFP comparing six reaction buffer compositions and three batches of independently prepared CFE. n = 3; error bars indicate standard error (s.e.m). D) Extracted responses of CFPS reactions performed with three batches (1, 2, 3) of CFE using six differing reaction buffers (Ref. 9, 19, 25, 33, 51). eGFP was used as the fluorescent reporter. n = 3; error bars represent standard error (s.e.m). E) Correlation of observed responses using Batch 1 CFE vs. responses predicted by Least Squares model. Linear regression analysis indicates significant correlation for ΔFEU and rate responses. n = 3; error bars represent standard error (s.e.m). See Fig. 4 Source Data.xlsx for reaction compositions and responses.

One of the key goals of CFPS optimisation is to identify conditions robust to variation in cell lysate composition. Cell lysates are typically ‘home-made’ and therefore are likely to introduce the most variability into the reactions. To confound this, it is also the component with the greatest impact on ΔFEU and on the maximum rate of reaction and is the component identified to interact with the most other components. Examination of results from across all experiments to date indicated that there was a large variation in CFPS from theoretically identical reactions, including a proportion of failed reactions (Appendix Fig. A2). This is in line with other work reporting poor reproducibility between identical reactions, even when maintaining the same reagents, site and operator [39]. We explored how variations in cell lysate may impact on the robustness of the reactions. We examined the performance of the reference composition and of five other compositions: these were buffer 33, which was close to the predicted best performing trade off mixture; buffer 9, which was predicted to perform in a similar manner to the reference; two compositions that are predicted to perform well across all three responses (25 and 51); and a mid-placed composition (19). We examined both the interactions of eight components of the buffer with the CFE as well as simulating the effect of pipetting error on assembling these compositions (Fig. 4A, B). The results indicate that, across each of the eight interactions, neither the reference composition, buffer 9 or buffer 33 offer a solution in which each of the three objectives are met. For buffer 9 and 33 it is principally a failure to achieve a short lag time, whereas buffers 19, 25 and 51 meet each of the objectives. We also simulated 5000 experimental runs for each of these compositions, allowing the concentrations of the nine reaction components to vary in line with permissible pipetting deviations for the epMotion® 5073 m. This analysis highlighted that, while buffers 25, 33 and 51 are predicted to perform well across the objectives, they may also be expected to demonstrate greater experimental variation than the other compositions (Fig. 4B). These three compositions contain the greatest volume of cell lysate, whereas buffer 19 (which meets each of the four objectives) has the advantage of using a third less cell extract than these reactions and is characterised by a smaller variation in simulated performance.

In addition to variability within a given cell lysate, we also tested how different extract preparations performed in these buffers. We prepared two new CFE batches from *E. coli* BL21-Rosetta™ 2 and analysed the time-course data for six buffers (Fig. 4C), which, through the extracted measurements (Fig. 4D), indicated noise within and between batches of CFE. Buffer 19 – while not performing at the top range – balances variation between CFE batches and, as a result, equivalent responses are observed in batches 1, 2 and 3. Reaction composition 9 also performs similarly between lysate preparations, however, the overall performance is considerably lower than buffer 19 (mean ΔFEU = 145.8 vs*.* 501.3, respectively). Although reaction buffers 33 and 51 achieve some of the highest responses (ΔFEU = 668.9 (batch 3) and 747.7 (batch 1) respectively), exceeding the top performance of buffer 19 (ΔFEU = 617.8 (batch 3)), both buffers also result in poor responses as low as ΔFEU = 167.7 (buffer 33, batch 1) and 0.32 (buffer 51, batch 3) illustrating the high level of variability resulting from these compositions. It is important to note that the reaction buffers containing lower CFE (buffers 9 and 19) exhibited less variation than those buffers with a greater proportion of CFE (buffers 25, 33 and 51). We conclude that reaction mixtures that minimise the amount of cell lysate create more robust environments for different preparations of cell extract. It is also noticeable that the ranked performance of CFE batches is not consistent between buffers. For example, batch 1 produced the strongest performances in buffers 9, 25 and 51 but the weakest performances in buffers 19 and 33. This suggests that CFPS performance is not solely dependent on lysate quality and that it is possible to overcome variation between lysate batches by balancing other reaction components. To further test the RSM, the predicted performance of each buffer was compared to the actual responses achieved with batch 1 CFE (Fig. 4E). The models give good prediction for ΔFEU, maximum rate and longevity. However, the rate lag proved more challenging to model further work may be needed to refine the model incorporating additional sources of variability.

### 3.5 Buffer performance is robust between different proteins and *E. coli* strains

**Fig. 5:**
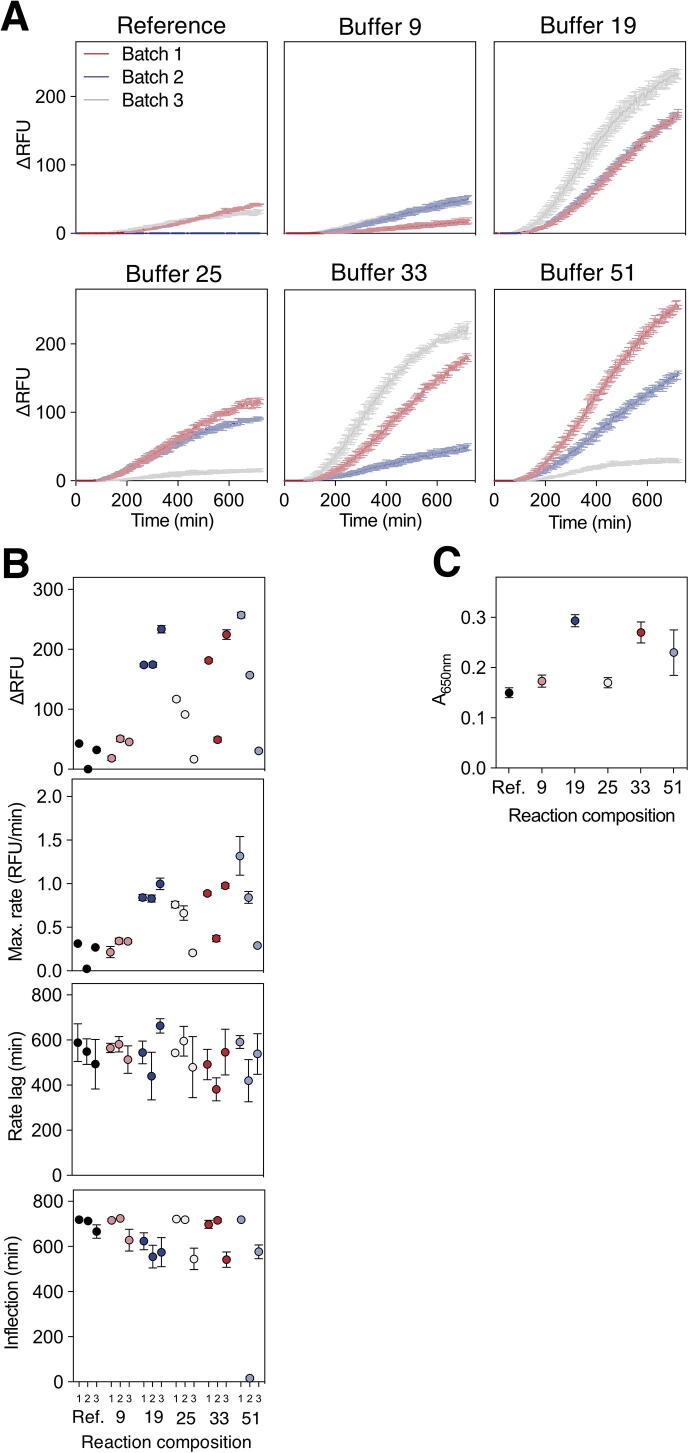
Validation of CFPS reaction buffers with synthesis of alternative proteins. A) Time-course data of CFPS reactions expressing mCherry comparing six reaction buffer compositions and three batches of independently prepared CFE. n = 3; error bars indicate standard error (s.e.m). See Fig. 5A and B Source Data.xlsx for reaction compositions and responses. B) Extracted responses of CFPS reactions performed with three batches of CFP using six differing reaction buffers. mCherry was used as the fluorescent reporter. n = 3; error bars represent standard error (s.e.m). See Fig. 5A and B Source Data.xlsx for reaction compositions and responses. C) Performance of six CFPS reaction buffers using CFP extracted from E. coli TOP10 and the pTU1-A-lacZ plasmid expressing β-galactosidase. X-gal (present at 1 mM) is metabolised by β-galactosidase resulting in a blue product detected at 650 nm. See Fig. 5C Source Data.xlsx for reaction compositions and responses. (For interpretation of the references to colour in this figure legend, the reader is referred to the web version of this article.)

For a CFPS buffer composition to be widely applicable it should be robust not only between different batches of CFE but also when synthesising different proteins and when using cell lysate prepared from different strains of *E. coli*. The six buffers tested using an eGFP template were consequently tested using equivalent template DNA coding for mCherry and with a template encoding LacZ⍺. For CFPS of mCherry, three batches of CFE prepared from *E. coli* BL21-Rosetta™ 2 cells were used (Fig. 5A, B). A similar pattern of results was obtained as for the synthesis of eGFP. Buffers 9 and 25 resulted in moderate responses, similar to the reference buffer, and buffers 19, 33 and 51 achieved a greater response. Similarly, greatest variation of performance between CFE batches was observed in buffers 25, 33 and 51, with minor variation between batches when using buffers 9 and 19.

Finally, the six different buffers were used in reactions containing a DNA template coding for the enzyme fragment LacZ⍺, required for ⍺-complementation of the β-galactosidase enzyme. Performance was quantified by detecting the presence of 5,5′-dibromo-4,4′-dichloro-indigo resulting from the hydrolysis of X-gal. CFPS reactions were performed using a CFE derived from *E. coli* TOP10 cells carrying the *lacZΔM15* mutation. The results for the synthesis of LacZ⍺ mirrored the pattern observed when testing the fluorescent reporters (Fig. 5C). Furthermore, the variability of reactions was also maintained, in that, buffers 33 and 51 exhibited more noise between replicates than the other buffers. Based on these results, we would recommend buffer 19 as a robust reaction buffer for *E. coli* lysate based CFPS as it maintains a strong, robust performance in the expression of different proteins and when using different batches of CFE as well as those derived from different *E. coli* strains.

## 4 Discussion

Cell-free systems provide a platform for the synthesis of target proteins outside the living cell. The open nature of these systems enables manipulation of the reaction environment to create conditions beyond those that would naturally occur. We have used a statistical engineering approach, guided by Design of Experiments (DoE) and data-driven modelling, to explore a large, multifactorial design space relating to the preparation of cell lysates and composition of CFPS reactions. In doing this, we have identified important reagents and key interactions which impact on various aspects of CFPS reaction kinetics. Furthermore, we identified a reaction buffer composition which has been demonstrated to perform robustly in the expression of different fluorescent reporter proteins and an active enzyme, as well as maintaining a robust performance between different batches of cell lysate of the same strain and across lysate preparations derived from different strains of *E. coli*. Reaction productivity improved substantially, compared to reference reactions, when using the alternative buffer composition. For eGFP and mCherry, ΔFEU and maximum reaction rate increased 2.5-fold and 4-fold respectively, although rate lag time and reaction longevity remained similar. In the expression of LacZ⍺, using *E. coli* TOP10 lysate, the response in the new buffer outperformed the reference reactions 2-fold.

Many of our findings agree with previously documented results. Magnesium is widely recognised as an important biochemical cofactor for many enzymes to function correctly. It is, therefore, not surprising that Mg-glutamate was consistently detected as having an important impact on CFPS performance, particularly with regard to ΔFEU and reaction rate. An interaction between Mg-glutamate and PEG-8000 was identified in the DSD and corroborated by the RSM analysis. This indicated that an increased Mg-glutamate concentration improved productivity when PEG-8000 concentrations were lower but could reduce the response when PEG-8000 concentrations were high. Nagaraj et al. have also reported the benefit of increased magnesium concentrations in CFPS reactions, particularly in reversing the translational inhibition that can result from high concentrations of NTPs [40]. Our analysis did not detect an interaction between Mg-glutamate and NTPs. In our investigations, however, the NTPs were treated as four independent factors in the DSD analysis (across a smaller concentration range) and fixed at a constant setting during the RSM. Our results did, however, agree with Nagaraj et al. that there is curvature associated with increasing Mg-glutamate concentration, and beyond 15 mM additional Mg-glutamate no longer benefits reactions and can in fact have a detrimental effect [40]. PEG is known to be a critical component in cell-free systems, playing a role in macromolecular crowding to enhance intermolecular associations. Our results agree with previous findings that, although there is a benefit to including PEG, high concentrations can negatively impact reaction performance [41], [42], [43]. This was most apparent when considering reaction rate – curvature was detected in the DSD, and the RSM also identified a significant inverse interaction between PEG-8000 and CFE suggesting protein concentration in the CFE may have comparable crowding effects to the those contributed by PEG. Our analyses also detected several components which did not have a strong impact on CFPS performance. In agreement with Borkowski et al. we found that varying the concentration of tRNA and CoA had little impact on performance, as such, these components were included in all reactions at a fixed setting but were not investigated beyond our screening experiments [23]. Borkowski et al. also observed that NAD, cAMP and folinic acid did not significantly impact yield. Likewise, we made the same observation from the results of the more in-depth RSM [40]. Although these components were not found to be key drivers of CFPS, reaction performance was diminished when they were removed which suggests they are involved in more subtle interactions.

Our investigations also revealed a previously unidentified relationship between 3-PGA and folinic acid. 3-PGA is present in reactions for ATP regeneration and folinic acid is required for translation initiation. If both components are included at a high setting, or both included at a low setting, the reactions do not perform well, however, it is possible to include both components if the concentrations are balanced i.e., if one is set high and the other low. This inverse interaction was found to significantly impact both the ΔFEU and rate responses. A possible reason this interaction has not been identified in previous studies may be that phosphoenolpyruvate (PEP) has historically been used for energy regeneration in cell-free systems. Glucose-6-phosphate (G-6-P) has also been used successfully for ATP regeneration producing a slower, but more sustained, reaction resulting in a higher protein yield overall than reactions using PEP [44]. 3-PGA has only been used as an alternative in more recent studies, although it has been reported to result in higher protein production compared to reactions using alternative ATP regeneration systems [45]. As a result, optimisation attempts using protocols requiring PEP or G-6-P will not have detected this interaction with folinic acid. A further, somewhat surprising result was that reactions were viable following the removal of many individual components. Indeed, it was even possible to remove combinations of multiple components and CFPS remained possible, albeit at a reduced level. This suggests that CFPS reactions are highly amenable to manipulation, and in certain settings it may be beneficial for the operator to remove components to reduce complexity or cost even if there is a trade-off against performance.

Robustness is a highly desirable characteristic of cell-free reactions, and the variability of these systems is widely acknowledged. Indeed, previous efforts have sought to reduce this variability by addressing lysate preparation [32]; identifying sources of variability associated with site and operator [39]; and recommending protocol modifications [46]. Despite these efforts, the composition of the reaction buffer itself has not been explored in detail as a means of reducing variability in CFPS performance. Our investigations revealed a novel buffer composition (buffer 19) that supports strong CFPS reactions, and also results in low levels of variation between replicates. Importantly, this buffer performs robustly between independently prepared batches of cell lysate. Critically, the fact that batch-to-batch variation was observed with some reaction buffers but not all suggests that this variation is not down to the CFE preparation method alone. This agrees with findings from Cole et al. who found that reagent preparation contributed significantly to performance variation but extract preparation method did not [39]. We noted that although some buffer compositions containing a high proportion of CFE resulted in a performance exceeding that of buffer 19, these typically exhibited greater variation between replicates and did not perform reliably in all batches of CFE. We propose that the composition of buffer 19 is sufficient to mitigate against the uncontrollable variation resulting from the biological component of cell-free reactions, when CFE is present at a moderate concentration of 10 mg/mL. Furthermore, buffer 19 also performed reliably when tested with DNA templates coding for different fluorescent reporter proteins, and when expressing LacZ⍺ using CFE derived from *E. coli* TOP10. Different *E. coli* strains may be required depending on the application of the cell-free system so it is, therefore, important to use a buffer composition that will behave robustly in combination with lysates derived from different strains.

By applying the principles of DoE to this work we have been able to explore the intricacies of a highly complex system. The use of statistically structured experiments ensured design spaces were explored in an efficient manner and removed the bias associated with OFAT experimentation. This approach complements the work of Nagaraj et al. who successfully modelled the impact of interacting reaction components on translation kinetics [40]. Similarly, the active-learning approach adopted by Borkowski et al. [23] was also effective in elucidating critical parameters impacting CFPS productivity. Although these studies explored buffer composition, our investigations encompassed a greater proportion of reaction components and a wider range of reagent concentrations, including attempts to remove entirely those components which were deemed to have a negative or non-significant impact on performance. Moreover, by considering multiple responses in parallel, we have been able to characterise the reaction components influencing different aspects of CFPS reaction kinetics.

## 5 Data statement

All data are available both in Source Data files associated with this publication, and at https://doi.org/10.25405/data.ncl.17041931.

## CRediT authorship contribution statement

**Alice M. Banks:** Methodology, Validation, Formal analysis, Investigation, Writing – original draft, Writing – review & editing, Visualization. **Colette J. Whitfield:** Investigation, Writing – review & editing. **Steven R. Brown:** Software, Investigation, Resources, Writing – review & editing. **David A. Fulton:** Conceptualization, Writing – review & editing, Funding acquisition. **Sarah A. Goodchild:** Conceptualization, Writing – review & editing, Funding acquisition. **Christopher Grant:** Software, Resources, Writing – review & editing. **John Love:** Conceptualization, Writing – review & editing, Funding acquisition. **Dennis W. Lendrem:** Formal analysis, Writing – review & editing. **Jonathan E. Fieldsend:** Conceptualization, Writing – review & editing, Funding acquisition. **Thomas P. Howard:** Conceptualization, Methodology, Validation, Formal analysis, Writing – original draft, Writing – review & editing, Visualization, Supervision, Project administration, Funding acquisition.

## Declaration of Competing Interest

The authors declare that they have no known competing financial interests or personal relationships that could have appeared to influence the work reported in this paper.
