# Supplementary material for: Key reaction components affect the kinetics and performance robustness of cell-free protein synthesis reactions
Source: Comput Struct Biotechnol J. 2021 Dec 13;20:218–29. doi: 10.1016/j.csbj.2021.12.013 (PMC8718664; doi:10.1016/j.csbj.2021.12.013)
Supplement: Supplementary data 1 [file mmc1.docx]

**Appendix**

***Method A1 Scoping trial for cell-free extract preparation and CFPS execution***

Cell-free extracts from *E. coli* BL21-Rosetta­^TM^ 2 were prepared using the method described by Sun *et al* with the modifications described in **Table A1** [1]. Concentrations of Mg, K and DTT were optimised for each preparation of cell-free extract using the buffer calibration protocol described by Sun et al. All reactions were performed at 50 µL volumes in black 384-well flat-bottomed microplates. CFPS reactions were incubated using a Varioskan LUX Multimode Microplate Reader (Thermo Scientific). A kinetic loop was used to record top-down fluorescence readings at 5-min intervals for a total of 4 h with continuous shaking at 60 rpm and incubation at 37 °C. eGFP production was monitored using an excitation of 488 nm and emission of 512 nm. An excitation bandwidth of 5 nm and measurement time of 100 ms was used for all readings. The final reaction conditions for most scoping reactions were: 8.9 mg/mL cell-free protein, 4.5-10.5 mM Mg-glutamate, 40-160 mM K-glutamate, 0.33-3.00 mM DTT, 1.5 mM each amino acid except leucine, 1.25 mM leucine, 50 mM HEPES, 1.5 mM ATP, 1.5 mM GTP, 0.9 mM CTP, 0.9 mM UTP, 0.2 mg/mL tRNA, 0.26 mM CoA, 0.33 mM NAD, 0.75 mM cAMP, 0.068 mM folinic acid, 1 mM spermidine, 30 mM 3-PGA, 2 % PEG-8000, 20 mg/mL pTU-1A_J23100_pET-RBS_eGFP_BBa_B0015. The exceptions to this were the low and high settings for the CFPS reaction buffer scoping reactions, which were 0.5 and 1.5 times the described concentrations respectively.

***Method A2*** ***Execution of Definitive Screening Designs in Fig 2A using the TTP Labtech dragonfly discovery and Antha (Synthace Ltd.)***

The TTP Labtech dragonfly discovery was used for the rapid set-up of DSD1 and DSD2 (Fig 2A). **Source Data** files contains the experimental design files for each of the respective DSD experiments (*Figure 2A DSD1 design.xlsx* and *Figure 2A DSD2 design.xlsx*), as well as the respective Antha software execution files (*Figure 2A DSD1 workflow.json* and *Figure 2A DSD2 workflow.json*) detailing parameter set points for automated experiment execution. These method files allow the DSD experiments to be repeated using the Antha platform.

***Table A1 Modifications to cell-free extract preparation and CFPS reaction composition.*** Scoping trial modifications made to the preparation of cell-free extracts and the set-up of CFPS reactions.

| **Scoping trial** | **Modifications to cell-free extract preparation and CFPS reaction** |
| --- | --- |
| Cell growth – Low | Mini-culture 1 was prepared by inoculating 2 mL 2xYT+P in a 50 mL conical centrifuge tube with a single *E. coli*colony and incubating at 37 °C, 150 rpm for 4 hr. Following incubation, mini-culture 2 was prepared by transferring 50 µL of mini-culture 1 to 25 mL 2xYT+P in a 250 mL conical flask and incubating as before. A large culture was prepared by transferring 100 µL of mini-culture 2 to 100 mL 2xYT+P in a 250 mL conical flask and incubating until the OD_600_ = 1.0.  Culture was transferred to pre-chilled 50 mL conical centrifuge tubes and cells pelleted by centrifugation at 1,500 g for 5 min at 4 °C. Cells were washed twice in 50 mL S30A buffer and centrifuged as before. Cell pellets were flash frozen in liquid nitrogen and stored at -80 °C. Cell pellet resuspension, cell lysis, extract clarification and CFPS reactions were performed as described by Sun *et al* [1]. |
| Cell growth – Moderate | Mini-culture 1 was prepared by inoculating 4 mL 2xYT+P in a 50 mL conical centrifuge tube with a single *E. coli*colony and incubating at 37 °C, 200 rpm for 6 hr. Following incubation, mini-culture 2 was prepared by transferring 100 µL of mini-culture 1 to 50 mL 2xYT+P in a 250 mL conical flask and incubating as before. A large culture was prepared by transferring 4.5 mL of mini-culture 2 to 450 mL 2xYT+P in a 2 L conical flask and incubating until the OD_600_ = 2.0.  Culture was transferred to pre-chilled 50 mL conical centrifuge tubes and cells pelleted by centrifugation at 3,000 g for 10 min at 4 °C. Cells were washed twice in 50 mL S30A buffer and centrifuged as before. Cell pellets were flash frozen in liquid nitrogen and stored at -80 °C. Cell pellet resuspension, cell lysis, extract clarification and CFPS reactions were performed as described by Sun *et al* [1]. |
| Cell growth – High | Mini-culture 1 was prepared by inoculating 6 mL 2xYT+P in a 50 mL conical centrifuge tube with a single *E. coli*colony and incubating at 37 °C, 250 rpm for 8 hr. Following incubation, mini-culture 2 was prepared by transferring 150 µL of mini-culture 1 to 75 mL 2xYT+P in a 250 mL conical flask and incubating as before. A large culture was prepared by transferring 40 mL of mini-culture 2 to 1 L 2xYT+P in a 4 L conical flask and incubating until the OD_600_ = 3.0.  Culture was transferred to pre-chilled 50 mL conical centrifuge tubes and cells pelleted by centrifugation at 4,500 g for 15 min at 4 °C. Cells were washed twice in 50 mL S30A buffer and centrifuged as before. Cell pellets were flash frozen in liquid nitrogen and stored at -80 °C. Cell pellet resuspension, cell lysis, extract clarification and CFPS reactions were performed as described by Sun *et al* [1]. |
| Bead beating – Low | Cell pellets were prepared using *Cell growth – High*conditions. Cell pellets were resuspended at a ratio of 0.8 mL S30A buffer per g cells. 4 g of 0.1 mm glass beads were added per g cells and the suspension vortexed to achieve a homogenous paste. This was transferred to bead beating tubes and disrupted for 30 s at 10 Hz. The bead-cell solution was transferred to micro-chromatography columns and centrifuged at 1,500 g for 2 min at 4 °C to separate cell lysate from glass beads. The lysate was transferred to fresh 1.5 mL microcentrifuge tubes and centrifuged at 10,000 g for 5 min at 4 °C to remove cell debris. Extract clarification and CFPS reactions were performed as described by Sun *et al* [1]. |
| Bead beating – Moderate | Cell pellets were prepared using *Cell growth – High*conditions. Cell pellets were resuspended at a ratio of 1 mL S30A buffer per g cells. 5 g of 0.1 mm glass beads were added per g cells and the suspension vortexed to achieve a homogenous paste. This was transferred to bead beating tubes and disrupted for 45 s at 20 Hz. The bead-cell solution was transferred to micro-chromatography columns and centrifuged at 3,000 g for 6 min at 4 °C to separate cell lysate from glass beads. The lysate was transferred to fresh 1.5 mL microcentrifuge tubes and centrifuged at 15,000 g for 10 min at 4 °C to remove cell debris. Extract clarification and CFPS reactions were performed as described by Sun *et al* [1]. |
| Bead beating - High | Cell pellets were prepared using *Cell growth – High*conditions. Cell pellets were resuspended at a ratio of 1.2 mL S30A buffer per g cells. 6 g of 0.1 mm glass beads were added per g cells and the suspension vortexed to achieve a homogenous paste. This was transferred to bead beating tubes and disrupted for 60 s at 30 Hz. The bead-cell solution was transferred to micro-chromatography columns and centrifuged at 4,500 g for 10 min at 4 °C to separate cell lysate from glass beads. The lysate was transferred to fresh 1.5 mL microcentrifuge tubes and centrifuged at 20,000 g for 15 min at 4 °C to remove cell debris. Extract clarification and CFPS reactions were performed as described by Sun *et al* [1]. |
| Sonication – Low | Cell pellets were prepared using *Cell growth – High*conditions. Cell pellets were resuspended at a ratio of 0.8 mL S30A buffer per g cells. Cell suspensions were sonicated at 120 W, 20 kHz, 30 % amplitude and pulsed 10s/10s on/off for 1 cycle. Tubes were centrifuged at 10,000 g for 5 min at 4 °C to pellet cell debris. Extract clarification and CFPS reactions were performed as described by Sun *et al* [1]. |
| Sonication – Moderate | Cell pellets were prepared using *Cell growth – High*conditions. Cell pellets were resuspended at a ratio of 1 mL S30A buffer per g cells. Cell suspensions were sonicated at 120 W, 20 kHz, 30 % amplitude and pulsed 20s/20s on/off for 5 cycles. Tubes were centrifuged at 15,000 g for 10 min at 4 °C to pellet cell debris. Extract clarification and CFPS reactions were performed as described by Sun *et al* [1]. |
| Sonication – High | Cell pellets were prepared using *Cell growth – High*conditions. Cell pellets were resuspended at a ratio of 1.2 mL S30A buffer per g cells. Cell suspensions were sonicated at 120 W, 20 kHz, 30 % amplitude and pulsed 30s/30s on/off for 10 cycles. Tubes were centrifuged at 10,000 g for 15 min at 4 °C to pellet cell debris. Extract clarification and CFPS reactions were performed as described by Sun *et al* [1]. |
| Extract clarification – Low | Cell lysates were prepared using *Cell growth – High*and *Cell lysis - High*conditions. The lysate was incubated in a run-off reaction performed at 37 °C, 150 rpm for 30 min. Following this, the lysate was centrifuged at 10,000 g for 5 min at 4 °C and the supernatant transferred to a hydrated 10k MWCO dialysis cassette. Dialysis was carried out in S30B buffer for 1 hr at 4 °C. After dialysis, the lysate was centrifuged at 10,000 g for 5 min at 4 °C and the supernatant transferred to a fresh microcentrifuge tube and stored at -80 °C. CFPS reactions were performed as described by Sun *et al* [1]. |
| Extract clarification – Moderate | Cell lysates were prepared using *Cell growth – High*and *Cell lysis - High*conditions. The lysate was incubated in a run-off reaction performed at 37 °C, 200 rpm for 60 min. Following this, the lysate was centrifuged at 15,000 g for 10 min at 4 °C and the supernatant transferred to a hydrated 10k MWCO dialysis cassette. Dialysis was carried out in S30B buffer for 2 hr at 4 °C. After dialysis, the lysate was centrifuged at 15,000 g for 10 min at 4 °C and the supernatant transferred to a fresh microcentrifuge tube and stored at -80 °C. CFPS reactions were performed as described by Sun *et al* [1]. |
| Extract clarification – High | Cell lysates were prepared using *Cell growth – High*and *Cell lysis - High*conditions. The lysate was incubated in a run-off reaction performed at 37 °C, 250 rpm for 90 min. Following this, the lysate was centrifuged at 20,000 g for 15 min at 4 °C and the supernatant transferred to a hydrated 10k MWCO dialysis cassette. Dialysis was carried out in S30B buffer for 3 hr at 4 °C. After dialysis, the lysate was centrifuged at 20,000 g for 15 min at 4 °C and the supernatant transferred to a fresh microcentrifuge tube and stored at -80 °C. CFPS reactions were performed as described by Sun *et al* [1]. |
| CFPS buffer composition – Low | Cell-free extracts were prepared using*Cell growth – High*, *Cell lysis – High*and *Extract clarification – Moderate*conditions. CFPS reactions were performed at the following conditions: 4.45 mg/mL cell-free protein, 2.5 mM Mg-glutamate, 70 mM K-glutamate, 0.75 mM DTT, 0.75 mM each amino acid except leucine, 0.625 mM leucine, 25 mM HEPES, 0.75 mM ATP, 0.75 mM GTP, 0.45 mM CTP, 0.45 mM UTP, 0.1 mg/mL tRNA, 0.13 mM CoA, 0.165 mM NAD, 0.375 mM cAMP, 0.034 mM folinic acid, 0.5 mM spermidine, 15 mM 3-PGA, 1 % PEG-8000, 10 mg/mL pTU-1A_J23100_pET-RBS_eGFP_BBa_B0015. |
| CFPS buffer composition – Moderate | Cell-free extracts were prepared using*Cell growth – High*, *Cell lysis – High*and *Extract clarification – Moderate*conditions. CFPS reactions were performed at the following conditions: 4.45 mg/mL cell-free protein, 5 mM Mg-glutamate, 140 mM K-glutamate, 1.5 mM DTT, 1.5 mM each amino acid except leucine, 1.25 mM leucine, 50 mM HEPES, 1.5 mM ATP, 1.5 mM GTP, 0.9 mM CTP, 0.9 mM UTP, 0.2 mg/mL tRNA, 0.26 mM CoA, 0.33 mM NAD, 0.75 mM cAMP, 0.068 mM folinic acid, 1 mM spermidine, 30 mM 3-PGA, 2 % PEG-8000, 20 mg/mL pTU-1A_J23100_pET-RBS_eGFP_BBa_B0015. |
| CFPS buffer composition – High | Cell-free extracts were prepared using*Cell growth – High*, *Cell lysis – High*and *Extract clarification – Moderate*conditions. CFPS reactions were performed at the following conditions: 4.45 mg/mL cell-free protein, 7.5 mM Mg-glutamate, 21 mM K-glutamate, 2.25 mM DTT, 2.25 mM each amino acid except leucine, 1.875 mM leucine, 75 mM HEPES, 2.25 mM ATP, 2.25 mM GTP, 1.35.45 mM CTP, 1.35 mM UTP, 0.3 mg/mL tRNA, 0.39 mM CoA, 0.495 mM NAD, 1.125 mM cAMP, 0.102 mM folinic acid, 1.5 mM spermidine, 45 mM 3-PGA, 3 % PEG-8000, 30 mg/mL pTU-1A_J23100_pET-RBS_eGFP_BBa_B0015. |

**
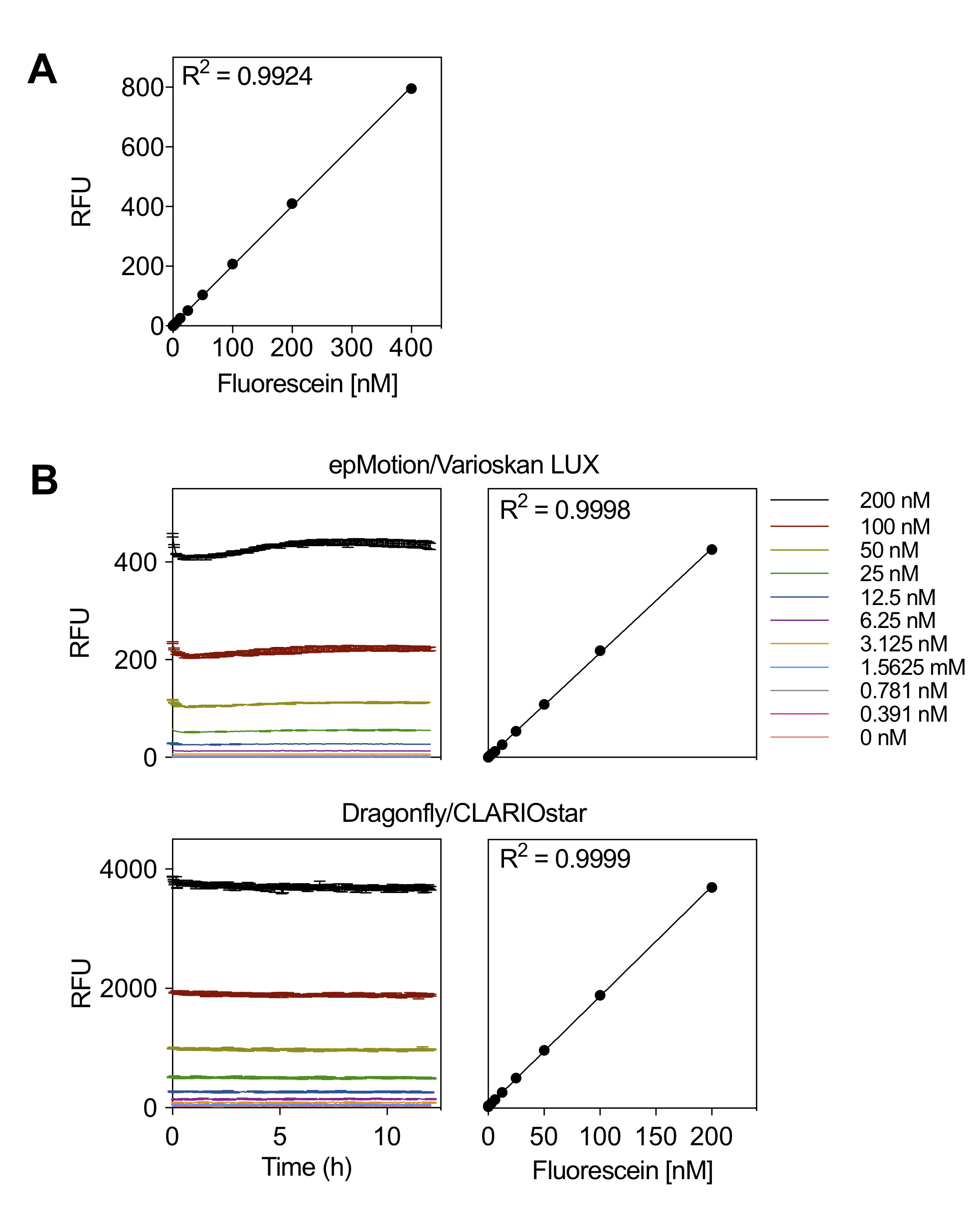
**

***Figure A1 Fluorescein standards.* A**) Linear relationship between fluorescein concentration and relative fluorescence (RFU). Tested over 20 independent runs performed using the epMotion for experimental set-up and Varioskan LUX for data collection. *n* = 20; error bars represent standard error (s.e.m). **B**) Relative fluorescence of fluorescein standards set up on the epMotion or dragonfly discovery and monitored for 12 h on the Varioskan LUX or CLARIOstar respectively (left). Linear regression of mean RFU at a range of fluorescein concentrations. *n* = 3; error bars represent standard error (s.e.m) (right). See *Figure A1 Source Data.xlsx* for fluorescein responses.

**
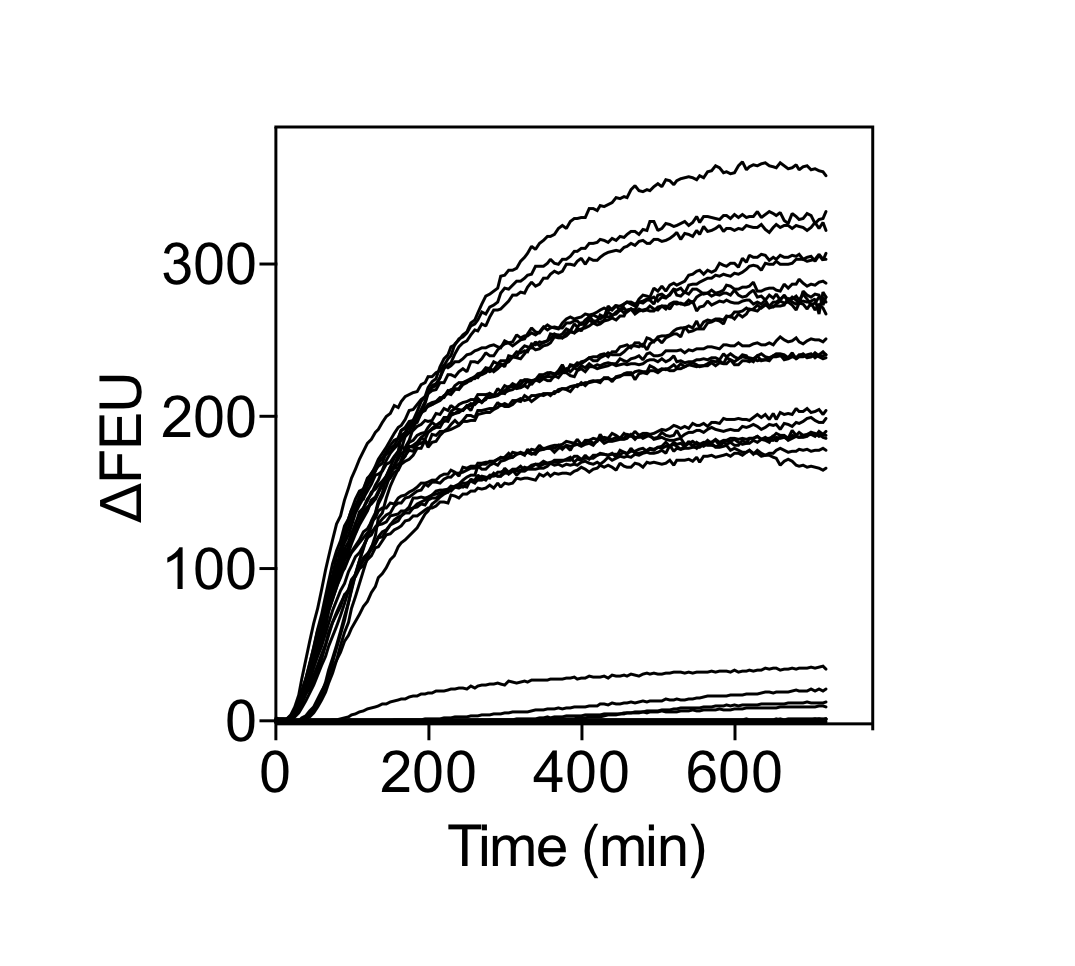
**

***Figure A2 Variation between independent CFPS reactions at the reference settings.*** Variation in CFPS reaction kinetics observed across 30 independent CFPS reactions using the reference reaction composition**.** See *Figure A2 Source Data.xlsx* for reaction compositions and responses.

**References**

[1] Sun ZZ, Hayes CA, Shin J, Caschera F, Murray RM, Noireaux V. Protocols for implementing an *Escherichia coli* based TX-TL cell-free expression system for synthetic biology. J Vis Exp 2013:e50762. https://doi.org/10.3791/50762.
