# Supplementary material for: Key reaction components affect the kinetics and performance robustness of cell-free protein synthesis reactions
Source: Comput Struct Biotechnol J. 2021 Dec 13;20:218–29. doi: 10.1016/j.csbj.2021.12.013 (PMC8718664; doi:10.1016/j.csbj.2021.12.013)
Supplement: Supplementary data 3 [file mmc3.docx]

**Supplementary Data**

***Fig S1. Impact of cell-free extract preparation and reaction composition on CFPS performance.*** CFPS reaction kinetics resulting from low, moderate and high settings of the three phases of CFE preparation (cell growth, cell lysis and extract clarification) and CFPS reaction composition (see **Appendix Method A1** and **Appendix Table A1** for details of CFE preparation and experimental settings). Moderate settings of cell growth, bead beating, sonication and extract clarification were performed in duplicate; error bars indicate standard error (s.e.m). Low and high settings of CFPS composition were performed in triplicate and moderate settings were performed in sextuplicate; error bars indicate standard error (s.e.m). See *Figure S1 Source Data.xlsx* for experimental conditions and responses.

***Fig S2. Effect of protease inhibitor, HEPES and 3-PGA on CFPS reaction kinetics.* A**) Effect of protease inhibitor concentration on ΔFEU. Mean ΔFEU of DSD1 reactions at three concentrations of protease inhibitor (concentrations of other reaction components vary). *n* = 3; error bars indicate standard error (s.e.m). Effect of HEPES pH on ΔFEU. Mean ΔFEU of DSD1 and DSD2 reactions at four pH levels of HEPES (concentrations of other reaction components vary). *n* = 22; error bars indicate standard error (s.e.m). See *Figure 2A and 2B Source Data.xlsx* for reaction compositions and responses. **B**) Drop-out tests screening for the impact of 3-PGA on CFPS kinetics. Drop-out tests to screen for essentiality and dose-dependent effects of 3-PGA. All other components are maintained at reference settings. *n* = 3; error bars indicate standard error (s.e.m). See *Figure S2 Source Data.xlsx* for reaction compositions and responses.

***Fig S3. Two-way interactions impacting ΔFEU, maximum rate, rate lag and inflection.*** Two-way interactions identified by the RSM impacting on ΔFEU. In each scenario all factors are fixed at the midpoint setting with the exception of the two interacting factors.

***Table S1. Literature search to identify common CFPS reagents and concentrations.*** Concentration ranges were used to determine factor settings used in DSD1 and DSD2.

*Table S1.xlsx*

***Table S2. Maximise Desirability settings for single and multi-objective optimisations.*** CFPS reaction component concentrations predicted by the Least Squares model based on RSM data to achieve optimal CFPS performance.

*Table S2.xlsx*
